# ‘You Can't Muck Around With Transplant’: Young People's Experiences of Clinical Care Following Lung Transplant

**DOI:** 10.1111/hex.70156

**Published:** 2025-01-28

**Authors:** Miranda Paraskeva, Hannah Gulline, Simone West, Louisa Walsh, Ben Tarrant, Kostas Hatzikiriakidis, Heather Morris, Darshini Ayton

**Affiliations:** ^1^ Department of Allergy, Immunology, and Respiratory Medicine Alfred Hospital Monash University Melbourne Australia; ^2^ Central Clinical School Faculty of Medicine, Nursing, and Health Sciences Monash University Melbourne Australia; ^3^ Health and Social Care Unit School of Public Health and Preventive Medicine, Faculty of Medicine, Nursing, and Health Sciences Monash University Melbourne Australia; ^4^ Department of Occupational Therapy Alfred Hospital Melbourne Australia; ^5^ Department of Nursing and Allied Health Centre for Health Communication and Participation Swinburne University of Technology, La Trobe University Melbourne Australia; ^6^ Department of Physiotherapy St Vincent's Hospital Melbourne Australia

**Keywords:** adolescent and young adult, healthcare, transplant

## Abstract

**Background:**

Lung transplantation improves survival and quality of life in young people with end‐stage lung disease. Few studies have investigated the clinical care experiences of young people after lung transplantation.

**Design:**

This qualitative study aimed to explore the experiences of young people who underwent lung transplantation. Semi‐structured interviews were conducted with 16 lung transplant recipients (< 25 years at transplant). Interviews were analysed to identify themes and categorize and describe the experience of young lung transplant recipients.

**Results:**

The themes that emerged were (1) Hope and spectre: The transplant dilemma; (2) Information delivery and comprehension; (3) Independence and navigating care; and (4) Continuity and youth‐appropriate care. Findings suggest that young people have distinct care needs that consider the many parallel life transitions that occur in addition to transplantation. They value consistent and familiar teams, which nurture autonomy and independence in the context of post‐transplant survivorship and highlight the importance of feeling that they can relate to the healthcare process.

**Conclusion:**

The results highlight key areas where adolescent lung transplant recipients can be supported by clinicians, enabling the development of youth‐friendly services that cater to this group's healthcare and psychosocial needs.

**Patient or Public Contribution:**

Sixteen lung transplant recipients participated in the study by completing a semi‐structured interview. Two additional lung transplant recipients who received lung transplants as adolescents and one parent of an adolescent lung transplant recipient participated in a Project Advisory Group (PAG) with six clinicians representing paediatric, adolescent, and adult healthcare experience. They provided advice on research design including the development and revision of the interview guide and recruitment methods. They additionally provided feedback on the preliminary findings and outline of the manuscript. A summary of results was presented to the PAG who in conjunction with the writing group developed a list of recommendations based on the themes identified and the tenets of youth‐appropriate care as set out by the World Health Organization. One lung transplant recipient was an author on the manuscript contributing to its writing and review before submission. The clinicians who participated in the PAG did not have direct healthcare relationships with the study participants.

## 1 Introduction

Solid organ transplantation (SOT) is a recognized and well‐developed treatment for end‐stage organ failure in adults and children. Over the last two decades, rates of SOT have increased [1] and short‐ and long‐term outcomes improved with advances in surgical techniques and medical therapies [2]. The number of children and young people undergoing SOT, in particular, has increased as paediatric SOT programmes have developed internationally [3]. In 2023, in the United States of America alone, more than 2000 SOTs were undertaken in children (based on OPTN data for Transplants in the United States as of December 11, 2024). While the majority of children and young people transplanted receive liver, kidney, or heart transplants, an increasing proportion are also receiving lung transplants [1, 3, 4].

Across all SOT groups, those transplanted during adolescence and young adulthood (10−24 years [5]) demonstrate poorer outcomes than younger children and older adults. Registry analyses of heart, lung, and kidney transplant adolescent and young adults (AYAs) have demonstrated increased mortality [6, 7, 8], as well as higher rates of acute and chronic rejection [9, 10]. Additionally, studies have demonstrated an increased burden of mental health disorders among AYA SOT recipients, including greater rates of anxiety, depression, and posttraumatic stress disorder [11, 12]. The reasons for the poorer outcomes in AYAs are as yet undefined but are likely influenced by the normative biological, psychological, and developmental transitions of adolescence, which potentially contribute to increased rates of nonadherence and increased risk‐taking.

Lung transplantation is a lifesaving therapy for children and young people with chronic lung disease. It increases life expectancy and improves health‐related quality of life [13]. While advances in lung transplant techniques have led to improvements in survival overall, similarly to other SOT groups, this has not been seen in those transplanted between the ages of 10−24 years [7]. Despite this, lung transplantation in the AYA offers the hope of a longer life without the limitations of respiratory illness. However, it comes with a significant cost to the individual, including a high burden of clinical care and medication regimens required across the life course [14], and is accompanied by significant adaptive challenges including developing the capacity to self‐manage a complex chronic illness, cope with side effects, and adjust to social and lifestyle limitations [15]. The model of clinical care following lung transplantation is based on the adult recipient and tends to follow a biomedical model, with a focus on physiological monitoring and self‐efficacy [16]. AYAs have different priorities and expectations of clinical care than older individuals valuing connection, flexibility, and autonomy, with a desire to centre their medical condition in the broader context of their life [17].

Despite the poorer outcomes seen in AYAs with a lung transplant, there is a lack of data pertaining to their lived experience and specifically what they value when it comes to healthcare. This study aimed to explore AYA experiences and perceptions of clinical care, with a view to understanding the journey of young individuals following lung transplantation and developing programmes that better meet their needs.

## 2 Materials and Methods

### 2.1 Study Design

A qualitative descriptive study with semi‐structured interviews was undertaken at the Alfred Hospital in Melbourne, Australia [18]. The study was approved by the Alfred Hospital ethics committee (Project No. 764/19). This paper is the second of three papers that looked at the experiences of young people following lung transplants and is reported in accordance with the Standards for Reporting Qualitative Research [19].

### 2.2 Participants

Purposive sampling was used to recruit lung or heart–lung transplant recipients aged 15−29 years who underwent transplantation at ≤ 25 years of age. Individuals (or their guardians) meeting inclusion criteria were contacted by telephone by a researcher not involved in their treatment. If they expressed interest in participating, study information was electronically provided. Guardian consent was obtained for those aged < 18 years at the time of the interview. The exclusion criteria were intellectual or learning disabilities impacting capacity and informed consent or being less than 6 months post‐transplant and medically vulnerable.

A total of 110 transplants were performed in 101 recipients aged ≤ 25 years between April 2006 and May 2020. At the time of the study, 61 recipients were alive and 31 met eligibility criteria and were invited to participate. Of those invited, 19 responses were received with 16 proceeding to participation (two declined due to instability in their health and one withdrew before the interview without disclosing the reason).

### 2.3 Project Advisory Group (PAG)

A PAG of six clinicians with experience in paediatric, adolescent, and adult healthcare, one carer, and two lung transplant recipients who were transplanted at age < 25 years was formed. The PAG provided advice on the design of the research, including the interview guide and recruitment, provided feedback on preliminary findings, and reviewed the manuscript before submission. A summary of the results was presented to the PAG who in conjunction with the writing group developed a list of recommendations based on the themes identified and the tenets of youth‐appropriate care as set out by the World Health Organization [17, 20]. The clinicians who participated in PAG were not known to the study participants.

### 2.4 Data Collection

Semi‐structured Interviews were conducted remotely via Zoom/telephone by one of four researchers (K.H., D.A., H.M., S.W.) between June and December 2020. Interviews followed a semi‐structured interview guide (Appendix SA). Interviews lasted between 37.77 and 91.67 min (mean = 57.24) and were audio‐recorded and transcribed for analysis.

### 2.5 Qualitative Analysis

Thematic analysis based on inductive and deductive coding to identify and categorize themes was used [18]. One researcher completed the analysis of all 16 transcripts (K.H.), four were chosen at random to double‐code by a second researcher (D.A.), and three researchers read all transcripts and reviewed the analysed themes (H.G., M.P., S.W.). Discussion led to a consensus agreement on themes and subthemes. All qualitative analysis was undertaken in NVivo version 20.3 (QSR International, 2022).

## 3 Results

Sixteen young people participated. The majority were female (*n* = 11) with an average age at transplant being 16 years. Participants resided in Victoria (*n* = 11) and other states of Australia (*n* = 5). Demographics are provided in Table 1.

**Table 1:** Demographic characteristics of young people who have undergone lung transplant.

Demographic characteristic (*n* = 16)	Descriptive statistics
Age (years): Mean (SD), range	
Age during the interview	22.6 (4.2), 16.1−28.6
Age during the transplant	16.4 (5.8), 6.8−25.3
Years since the transplant	6.2 (3.7), 1.0−14.3
Gender: *N* (%)	
Male	5 (31.2)
Female	11 (68.8)
Indication for the transplant: *N* (%)	
Cystic fibrosis	6 (37.5)
Pulmonary arterial hypertension	4 (25.0)
Other health conditions^a^	6 (37.5)

^a^ Other health conditions include congenital heart disease, interstitial lung disease, and obliterative bronchiolitis.

**Figure 1:**
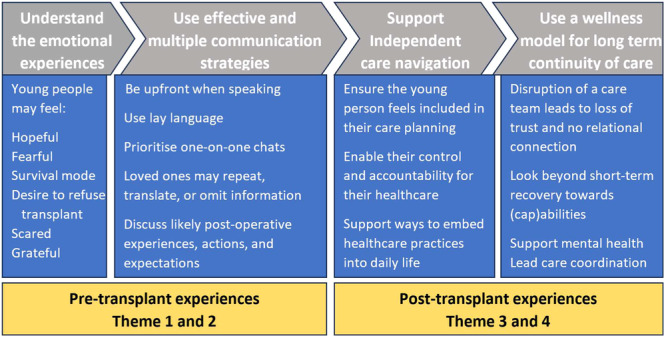
Overview of the outcomes and actions emerging from themes.

The following results reflect perceptions of lung transplant clinical care, as experienced by young people transplanted in childhood and adolescence. Thematic analysis generated four themes: (1) Hope and spectre: The transplant dilemma; (2) Information delivery and comprehension; (3) Independence and navigating care; and (4) Continuity and coordination of care. Bolded text within the narrative indicates a subtheme. Table 2 provides a summary of the themes with illustrative quotes. Figure 1 provides an overview of the outcomes and actions that have emerged from the themes.

**Table 2:** Overview of findings: Themes, subthemes, and examples.

Theme	Subtheme	Quotes
1. Hope and spectre: The transplant dilemma	Subtheme 1 Hope and anticipatory fear	I was pretty young then and didn't know what was ahead of me. (LTx1, F) You hear so many stories about CF that I was like, ‘Why would I want a transplant when I could potentially need another one?’, not realizing how different my disease was in a way that I don't have to live with my disease if I have a transplant. (LTx5, F)
	Subtheme 2 Only option for extended survival	I understood a little bit. If it didn't happen, I wouldn't have been able to survive for longer. (LTx13, M) I got re‐referred back to the transplant team and they pretty much said, ‘You are very sick and you're not going to get any better. To be honest, the lung transplant is your last option.’ I still kind of wanted to say no. (LTx9, F) It was the half and half. You don't know what's going to happen, so it's that terrifying bit that I may not survive or come out of it. (LTx13, M)
	Subtheme 3 Juxtaposing sentiments post‐transplant	People are always like, ‘Oh, you must feel so much better, you must be so happy and grateful’ blah, blah, blah. I am grateful but it's still really hard afterwards, even though I don't have pulmonary hypertension anymore. But now I have something else. It's not exactly an illness, but it is like something like a medical condition. It's still a lot of work and it still affects your life in a big way. (LTx4, F) I'm very glad and very grateful. The alternative is not so great. I'm very glad I had a transplant. There are moments, but they are very, very brief moments of doubt and so on. But overall, I'm over the moon about it. (LTx12, M) It's offered this longer lifeline for me, but on the other hand, I feel like it's a quality of life that sometimes is not so great. I've got my moments and days with it. Sometimes I'm happy for it and other times I'm like, ‘Ooo’… I always feel really bad too when I have to admit that I'm not 100% like, ‘Yeah! I'm happy about it’. But it's the reality. (LTx2, F)
2. Information delivery and comprehension	Subtheme 1 Content and style of communication	Nothing has really set me back where I've gone, ‘Oh shit, I wish that was explained better to me’. I honestly think that they did the best job they possibly could. I don't think they could have done much more in terms of preparing a 15‐16‐year‐old. (LTx12, M) I think they were pretty clear. There's not much good beating around the bush about it. (LTx8, F) Anything I didn't understand, they had really good ways of breaking it down, so it was digestible (LTx12, M) Go through it step by step, not all in one big go. (LTx15, F) I think having it light‐hearted enough to not scare someone, but at the same time, serious enough that they understand that you can't muck around with a transplant. (LTx12, M) The team at The *** pushed quality of life being the thing that they hope is the best outcome out of it…They've got to go through the risks of being immunocompromised, diabetes, all the things that go in‐hand with transplant. They didn't miss anything out that's for sure. (LTx12, M)
	Subtheme 2 Family as gatekeeper and interpreter	I did have what they said clarified afterwards. (LTx14, M) Plenty of information was given to my parents that if I did have queries, we could talk it through. (LTx12, M) My mum and dad knew about it, but they didn't tell me. (LTx1, F) I didn't know anything, and it was a bit scary. I wasn't really sure what was going on a lot of the time. But I got through it. (LTx10)
	Subtheme 3 Introduction to the possibility of transplant	Since I was fairly young, I did have a concept of it. (LTx13, M) I didn't know in its entirety what was involved, but I knew the basics of it. We were prepped at a very young age. (LTx12, M) I think I was about 9 when they started talking about it. (LTx11, F) I understood it as, you take out the old lungs, you pop the new ones in, a really simple procedure (LTx16, F)
	Subtheme 4 Managing expectations of life post‐transplant	Not quite knowing what it was like to have lungs that work how they were meant to work, I definitely felt a lot better than what I was expecting from the workup. (LTx12, M) The post information that you get is more than what you need – it's very thorough (LTx12, M) It went better than I was expecting. Obviously, they had to explain the worst‐case scenario type thing and things like that. I think it was pretty good. (LTx14, M) The initial hospital‐based recovery was easier than I thought it was going to be. It was the amount of complications I had afterwards that I hadn't put a lot of thought into. (LTx7, F) I wasn't expecting it to be as painful afterwards maybe. (LTx8, M)
3. Independence and navigating care	Subtheme 1 The challenges of navigating care	I've got a blue folder and it's got all my paperwork in it for the hospital and transplant information and stuff like that, because otherwise it gets a bit confusing. (LTx4, F) I guess the one other thing you don't realize is that hospitals are exhausting. (LTx7, F)
	Subtheme 2 Learning independence and self‐management	In the first year I definitely wanted someone there with me, but now I could do it by myself. (LTx4, F) You can't be impulsive…I definitively have to think things through before I make decisions. I guess that makes my decisions wiser. (LTx11, F) Probably from about six or seven, my parents never stayed the night. That was the thing, they never stayed. I was by myself. I grew up in the hospital in that process. (LTx6, M) There is no least important or most important; it's all important. I just think not taking on all the responsibility at once is something that is really, really helpful when you are starting to do your own treatments. (LTx8, F)
4. Continuity and youth appropriate care	Subtheme 1 Continuity of care team	Imagine seeing a different GP every time. That would drive you nuts, wouldn't it? you'd be like, ‘Oh, this person doesn't know me’ (LTx6, M). It was helpful to just communicate with one person (LTx5, F) So you've got the doctor that looked after me for the first four years and I've had a different doctor probably every time since… It's a bit of a joke if you ask me. What happens is I have to tell my story and explain it every time. (LTx6, M) My family reported being very thankful for the fact that I had the same few nurses looking after me continually because it allowed them to build that relationship with them and it made them feel more secure as well. (LTx7, F) I'm so happy that the transplant team were so lovely; I can't imagine another team. I've thought multiple times, ‘I want to move to this place’ but then my Mum is like, ‘How? You love the transplant team. Can you imagine adjusting to a whole other team?’ (LTx9, F)
	Subtheme 2 Coordinate care	It's really good in the transplant clinic that they have all the specialties there together, so you can see everyone all in the one place. (LTx7, F) Doctors have a big, big focus on medical recovery, not so much mental and physical ability post‐transplant. (LTx8, F)

Abbreviation: LTx, lung transplant recipient.

### 3.1 Theme 1: Hope and Spectre: The Transplant Dilemma

Many participants spoke about their emotional responses to learning that they needed a transplant. While most understood that this was aimed at improving their quality of life, they expressed mixed responses to that knowledge. **Anticipatory fear** was a common description, ‘I just remember [lung transplant] being the scariest thought at the time’ (LTx5, F).

Young people described understanding that they were going to die of their disease and that transplant was their **only option for extended survival**, ‘If it didn't happen, I wouldn't have been able to survive for longer’ (LTx13, M). Many felt they had no choice but despite knowing they were out of options, some were still resistant to the idea of transplantation and were tempted to refuse, ‘I still kind of wanted to say no’ (LTx9, F).

Young people may have been aware of their future need for transplant but lacked understanding of post‐transplant life, contributing to fear. They focused on the potential negative outcomes rather than **hope** for improvements in quality of life ‘why would I want a transplant when I could potentially need another one?’ (LTx5, F). Young people acknowledged that transplant is not a cure and that they were exchanging one disease for a lifelong commitment to medical care. When considering life after transplant, young people expressed the **juxtaposing sentiments** of being grateful to have received the gift of transplant and the burden of having the expectation of gratitude when they are faced with the burden of care, stating that there are times when it is a challenge to feel happy, ‘I am grateful but it's still really hard afterwards’ (LTx4, F).

### 3.2 Theme 2: Information Delivery and Comprehension

Young people described a wide variation of experiences and levels of understanding of transplant before the procedure. Explanations provided by the lung transplant medical team were identified as a key enabler to feeling informed about thr transplant. A specific skilled team approach to information sharing about the transplant was described as both useful and trusted. Most recipients described feeling comfortable with the information they were provided pre‐transplant.

**Information delivery and the tone** used to convey information were important to young people, and both impacted their understanding and experience of clinical care. Most participants preferred a realistic approach, valuing honest and transparent communication. They valued the treating team being ‘as upfront as possible’ (LTx8, M) and using clear language and concepts that were relatable and understandable ‘normal words and not like doctor words’ (LTx4, F). Participants acknowledged that what information was delivered and how it was presented needed to be age‐appropriate and tailored to the young person's maturity and capacity to cope. AYAs highlighted the need to break down complex concepts into manageable amounts to facilitate learning. They felt that while carers needed to be involved in discussions and education around transplant, the focus of clinicians should be on centreing the individual young person ‘Having a one‐on‐one chat …to make sure they actually understand the process of what is actually going on’ (LTx16, F). Overall, they expressed the importance of the transplant team finding a balance between being blunt yet sensitive, providing information in a structured yet relaxed manner—light‐hearted enough not to scare someone, but serious enough for them to understand.

Participants highlighted the role of **family both as a gatekeeper and an interpreter**. Young people who participated in medical consultations about the transplant with family described a more positive experience of learning about the transplant and, while still feeling apprehensive, did not necessarily feel overwhelmed. Many described a reliance upon family members to interpret concepts and language associated with transplant. ‘My parents would probably end up telling me in a more sensible manner and say something I could understand’ (LTx13, M).

In contrast to when family members acted as interpreters of transplant information, family members could also act as gatekeepers, by censoring or withholding information about the transplant ‘my mum and dad knew about it, but they didn't tell me’ (LTx1, F). Young people who felt that they needed to know more than what their parents had shared typically sought to independently learn about what may happen to them during a transplant, ‘I didn't know what a transplant was… I used my free periods [at school] to find out what a transplant meant’ (LTx1, F).

**Introduction to the possibility of transplant and its impact.** For those with life‐long chronic conditions (e.g., cystic fibrosis [CF]), early engagement of the young person and their family in concepts associated with transplant was more routine and for some initiated in childhood by their primary care team.

For those who experienced transplant as an emergency procedure without pre‐warning, they describe waking in ICU and learning of transplant, ‘It literally just happened… I was blindsided by it’ (LTx2, F). These young people described the challenges of coping with life after transplant and the resentment they felt toward the process ‘If you knew beforehand that you needed a transplant and you're on the list, then when you got it, you'd be like, “Wow this is great”. But if you don't know beforehand, it feels like the transplant is the reason why you are now going through this’ (LTx4, F). For these young people, the immediate support of bedside nurses and family assisted them in comprehending what had happened to them and what was to happen next. However, even for the young people who actively participated in the decision‐making to be listed for the lung transplant, there were still narratives of feeling under‐prepared for the challenge of recovery, the ongoing medical treatment necessary, and the complications they encountered.

**Managing expectations of life post‐transplant** was discussed by all participants. Despite most young people expressing satisfaction with the information given before transplant, when asked ‘was transplant what you expected?’ There were mixed responses. Some described outcomes exceeding their initial expectations, while others felt unprepared for the challenges of post‐transplant life. Participants reported that it was not the operation itself that proved the most challenging but the recovery phase and many did not appreciate the ongoing treatment required and potential complications. ‘The initial hospital‐based recovery was easier than I thought it was going to be. It was the amount of complications I had afterwards that I hadn't put a lot of thought into’ (LTx7, F). In particular, the complexities of hospital attendance, medication regimens, and medication side effects. ‘One that's not that bad, but it's just annoying is moon face—it's from the prednisone’ (LTx4, F).

Many young people described a preference for more information about postoperative experiences they may encounter, ‘You could have told me that beforehand’ (LTx4, F), with information targeted to their age and educational level. Yet others preferred basic fundamental information, ‘I only listened to the basics, I didn't want to get too overwhelmed’ (LTx5, F).

### 3.3 Theme 3: Independence and Navigating Care

Challenges of navigating care. Most young people described their care as person‐centred. This was characterized by the amount of ‘say’, control and inclusiveness in the provision of information, ‘I can't think of any instances where I feel that I wasn't included, or my views weren't accounted for’ (LTx7, F), and also their involvement in making health‐related decisions ‘I definitely have [a] say in it’ (LTx12, M). Feeling safe to offer an opinion and feeling respected was important to young people.

While the majority of young people believed they should be supported in attending appointments alone, they also described **the challenges of navigating care** and noted that, initially, they typically needed to rely on family for guidance and support. They expressed that as routines and clinicians became more familiar, their independence developed in parallel.

**Learning independence and self‐management** skills was a dynamic process for young people who described the constant need to attend appointments, organize medications, and complete pathology requests as challenging. Individual strategies to manage competing demands were skills acquired as part of the rehabilitation and transition programme to support young people in navigating routine care with optimal independence. Several young people shared the way they managed the complex regime of post‐transplant medication using dosettes or phone alarms.

Young people in this study generally reflected a high awareness of how to manage their health post‐transplant. Some attributed this awareness to their experience of the death of peers, ‘It's amazing how many people just think they're immortal. One of my good friends that has passed, he just thought, “This is my second chance and I deserve to just do whatever”’ (LTx6, M), describing an understanding that impulsive decisions could impact their health and the importance of forward planning, ‘say if I just go out for dinner, I'll always take my night dose of tablets, just in case, because you never know’ (LTx3, F).

### 3.4 Theme 4: Continuity and Youth Appropriate Care

For inpatient and outpatient care, a core team that was familiar and available when needed was described as an important feature of support, ‘It was helpful to just communicate with one person’ (LTx5, F). For those for whom continuity of post‐transplant care was disrupted with a loss of trust was reported, “Imagine seeing a different GP every time. That would drive you nuts, wouldn't it? You'd be like, ‘oh this person doesn't know me’” (LTx6, M). The lack of **continuity of care team** was magnified by a perceived mismatch in what medical staff and young people felt was important, ‘The doctors… have a big, big focus on medical recovery, not so much mental and physical ability post‐transplant’ (LTx9, F). In particular, AYAs felt that there needed to be more enquiry into their mental health with a focus on thquality of life ‘They don't really give much to that area I feel like, which I think they should’ (LTx4, F). One individual spoke of the traumatic nature of transplantation and the impact it had on their mental health. While clinicians did mention in passing that mental health support was available if needed, they did not take the initiative to coordinate care by asking if the young person needed such support (e.g., psychologist) ‘no one's really actually there saying, “Do you want to do that? Do you want me to make an appointment for you right now with this person?”’ (LTx4, F).

## 4 Discussion

Lung transplant is a chronic condition with significant treatment demands and long‐term risk for rejection, potential graft failure, and need for re‐transplantation [13]. While the outcomes of paediatric lung transplants continue to improve, AYAs (10−24 years) have overall worse outcomes than both younger children (< 10 years) and older adults [21]. This study, in examining the clinical care experience of AYAs following lung transplantation, highlights the need for strategies that differ from the traditional biomedical model that focuses on the physical sequelae of transplant.

AYAs expressed the importance of being regarded as a key player in their healthcare journey. Their relationships with healthcare professionals were integral to their clinical experience. Having a consistent team who respected their autonomy and included them in decision‐making was valued and engendered trust. This is consistent with other studies that highlight trust, autonomy, and shared decision‐making as critical in the development of successful healthcare relationships [22, 23, 24, 25]. Young people valued a team that regarded their transplant in the context of their life experience, taking into account their social and psychological functioning as well as the physiological monitoring of their allograft. This is a departure from the traditional biomedical model and its focus on diagnosis and treatment but is consistent with research that highlights the benefits of models of care that take into account social, environmental, and psychological factors and how these contribute to an individual's health [26]. The communication style was just as important as the content, with the key being the provision of age‐appropriate information in lay language.

Anticipatory fear characterized the period leading to transplant for many, and while participants highlighted the importance of pre‐transplant preparation and education in helping them manage time on the waitlist and post‐transplant life, many felt unprepared for the requirements and complications that they then faced. Some participants were tempted to refuse that transplantation has implications around informed consent, how alternative treatment pathways (including palliation) are discussed, and the rights of young people to refuse treatment. The care of young people requiring transplantation involves balancing both what is best for the AYA with the rights of the caregivers to control the narrative. Caregivers were at times described as gatekeepers of information, raising ethical issues around the balance of parental authority versus the needs of the adolescent minor [27]. Transplantation is a lifelong commitment and has far‐reaching implications on all aspects of a young person's life. Given that a number of studies have shown that young people aged > 14 years have decision‐making capacity and an understanding of death that is comparable to that of adults [28, 29, 30], their involvement in decisions about lung transplant should be encouraged with the provision of timely, clear, and age‐appropriate information.

AYAs described transplantation as physically and emotionally challenging. Like participants in other studies [23, 31], they described the dichotomy of their feelings following the transplant, being both grateful and resentful at times, especially in light of complications and disappointments when expectations were unmet [23, 32, 33]. The provision of information regarding transplantation was critical, and though the spectrum of what degree of information was desirable (ranging from ‘just the basics’ to more extensive), freely available information from a trusted source was imperative. This was highlighted both by individuals who underwent transplantation emergently without significant prior education and those who had information withheld from them by parents or healthcare providers. Studies have shown that AYAs feel fearful, anxious, and depersonalized when information is withheld or they are denied involvement in their own healthcare [24], highlighting both the critical need for tailored information and the challenges of meeting information demands in a world where young people derive much of their health information from nontraditional sources such as social media [34].

The roles of family in supporting individuals and interpreting information and experiences were highlighted by lung transplant recipients. Family participation was valued with parents/carers playing the role of both caretakers and gatekeepers of information. The importance of the inclusion of family in complex medical decision‐making has been highlighted in other studies and is in contrast to the perceived importance attributed to young people developing the skills to undertake lone consultations as part of their preparation for transitioning to adult healthcare [22]. AYAs viewed their family as a key source of support and, similar to other studies, expressed the importance of including family members while also emphasizing the importance of the young person having control over their presence and involvement [22]. Parental involvement and knowledge of medication regimens have been identified as playing an important role in adolescents' adherence to medical regimens and clinic attendance [35, 36] and should be supported by healthcare professionals.

Studies of adolescent liver and kidney transplant recipients have identified a range of psychosocial and adherence issues post‐transplant including depression, fear and anxiety, low self‐esteem, difficulties managing medication side effects, and the importance of family support [37]. However, very few studies have examined the information and clinical support needs of AYA SOT recipients [37, 38]. Those that did, however, similarly to our study, found a need for developmentally appropriate information with a specific focus on topics relevant to young people's lives and continued support for autonomy and development of independence.

We believe that this study provides insight into the clinical care needs of AYAs following transplantation and, by hearing the voices of young people, offers guidance on how to improve care in the clinical environment. Figure 1 and Table 3 summarize the recommendations developed by the PAG, which integrated the themes identified through this study and the principles of youth‐appropriate care [39].

**Table 3:** Recommendations for youth‐appropriate transplant care.

**Healthcare team** Consistent and familiar team. Encourage autonomy and shared decision‐making. Address social and psychological functioning as well as the physiological function of the allograft. Support participation of family but allow a young person to control their involvement. Ensure young people do not have healthcare information withheld from them.
**Communication style** Age‐appropriate information provision. Centre the young person. Use simple plain language.
**Information provision** Ensure that there is freely available information from a trusted source. Ensure that information is provided at intervals, in plain language. Review information over time as the individual ages and matures.

## 5 Limitations

This study was conducted at a large collocated paediatric and adult lung transplant centre, and findings may not reflect patient experiences at other centres as the majority of the cohort did not transition between paediatric and adult healthcare providers. Additionally, our participants were on average 6.2 years post‐transplant, and their retrospective recollection of what they felt, thought, and experienced may differ from those who are earlier in their transplant journey. Only English‐speaking participants were included, and thus, the additional care needs of culturally and linguistically diverse groups were not captured. Despite this, the findings were consistent with those of other studies on the care experiences of young people with other complex chronic diseases, including SOT. The interviews were conducted online during the COVID‐19 pandemic at a time when the state of Victoria was in a prolonged lockdown. While the changing nature of healthcare during the pandemic was noted by participants (and has previously been reported on) [40], the focus of this analysis was on healthcare interactions and the experience of young people accessing lung transplantation. Of note, none of the participants were assessed or transplanted during this period. Rather than a limitation, we believe this is a strength of the study, as it describes the healthcare needs of young people with a lung transplant in the current healthcare climate and highlights the importance of maintaining relationships and communication.

## 6 Conclusion

Increasingly, it is being recognized that AYAs accessing healthcare have unique needs [17] and, in particular, that the type and quality of care received by AYAs influence their adherence, self‐management, and engagement with healthcare providers [41]. A greater understanding of the experience of AYAs following lung transplants is crucial to inform the development of youth‐friendly services that cater to the healthcare and psychosocial needs of this vulnerable group. Figure 1 and Table 3 make suggestions for actions to support youth‐appropriate care following transplantation and include recommendations for the provision of consistent and familiar staff who respect the autonomy and agency of AYAs, as well as strategies to improve communication and foster independent self‐management over the long term. We suggest that strategies such as the ones recommended may lead to improved engagement with healthcare and given the poor outcomes and high rates of nonadherence in AYAs with SOT could in turn lead to improvement in outcomes.

## Author Contributions

**Miranda Paraskeva:** conceptualization, investigation, funding acquisition, writing–original draft, methodology, writing–review and editing, formal analysis, project administration, data curation, supervision. **Hannah Gulline:** writing–original draft, methodology, writing–review and editing, data curation. **Simone West:** conceptualization, investigation, writing–review and editing, resources. **Louisa Walsh:** conceptualization, writing–review and editing. **Ben Tarrant:** conceptualization, writing–review and editing. **Kostas Hatzikiriakidis:** conceptualization, investigation, writing–review and editing, writing–original draft, formal analysis. **Heather Morris:** conceptualization, investigation, writing–review and editing, formal analysis. **Darshini Ayton:** conceptualization, investigation, writing–review and editing, writing–original draft, supervision.

## Ethics Statement

The study was approved by The Alfred Hospital Ethics Committee, Melbourne, Australia (Project Number: 764/19).

## Conflicts of Interest

The authors declare no conflicts of interest.

## Supporting information

Supporting information.

## Data Availability

The data that support the findings of this study are available on request from the corresponding author. The data are not publicly available due to privacy or ethical restrictions.
