# Supplementary material for: ‘You Can't Muck Around With Transplant’: Young People's Experiences of Clinical Care Following Lung Transplant
Source: Health Expect. 2025 Jan 28;28(1):e70156. doi: 10.1111/hex.70156 (PMC11775388; doi:10.1111/hex.70156)
Supplement: Supplementary file 1 — Supporting information. [file HEX-28-e70156-s001.docx]

**Appendix A – Semi-structured Interview Guide**

Priority questions are highlighted in a bold font. Dot points indicate probing questions if needed.

| **Topic** | **Questions** |
| --- | --- |
| Transplant | How old were you when you had your transplant?  **How much did you know about what a transplant was before you had one?**  Who explained transplant to you?   - What did the doctor say? - What did your family say**?**   **Reflecting back, how much did you understand about what they were saying about the operation?**  Do you think they prepared you well enough for how you would feel after the operation?   - ICU - Ward - Physically - Emotionally   Was the transplant how they described it to you?  What things do you think you should have known more about before you had the transplant?  **How do you think young people should be taught about lung transplant?**  **If you were to explain what a lung transplant would be like to a young person who was thinking of having one, what would you say?** |
| After transplant: Processes of care | **Did you think you would have to keep coming to hospital after your transplant?**   - Does this interfere with school, work, friendships?   What do you think about all the medications you have to take?   - Do you understand what they are for and why you are taking them?   **What do you think about all the rules about what you can eat and do after transplant?**   - Medication regime - Diet - Exercise - Alcohol - Bloods and bronchoscopies - Tattoos/body piercing/manicures/pedicures - Self-efficacy   **On days when you don't feel well, do you get worried it has something to do with your transplant?**  Did you find your family were helpful after transplant?   - Did they help to explain things that people were saying to you that didn’t make sense? - Did they make you feel safe? - Did they come with you to appointments or procedures? - Did they need to help you with day-to-day things?   **Do you think you have enough say in things that affect your health?**   - Person centred care - Collaborative decision making - mutual respect   Do you think young people should be able to go to appointments without their family coming too?  How do you think hospital appointments and important information about you could be better? |
| After transplant: Everyday life | **What is good about the life you have?**  What is not so good?  How has having a transplant affected your life choices?   - Living independently - Relationships - Sex - Study - Work - Travel - Being a parent   Do you feel your health affects others? If yes, in what ways?   - Parents/siblings/partners   How has your transplant affected your ability to have relationships with others?   - Intimacy - Body image (scars, feeding tubes [PEGS], cushingoid features from steroids) - Gastric issues   **Are you glad you had a transplant? Yes/no (please tell us why)**  Where do you see yourself in 3 years’ time? |
| After transplant: Life considerations/ Social-emotional wellbeing | **How many other young people do you know who have had a transplant?**   - Has the health of friends with transplants affected them? - How important do you think it is to interact with other young people who have had a transplant?   **Who do you turn to when you need to talk with someone about your life and transplant?**  **If you could tell a young person 2 important things about life after transplant, what would they be?** |
